# Reminiscences of a European Trip to the International Medical Congress at Moscow, Russia, August 19 to 26, 1897, as a Delegate from the American Medical Association and the Odontological Society of Pennsylvania

**Published:** 1898-11

**Authors:** W. G. A. Bonwill

**Affiliations:** Philadelphia


### REMINISCENCES OF A EUROPEAN TRIP TO THE INTERNATIONAL MEDICAL CONGRESS AT MOSCOW, RUSSIA, AUGUST 19 TO 26, 1897, AS A DELEGATE FROM THE AMERICAN MEDICAL ASSOCIATION AND THE ODONTOLOGICAL SOCIETY OF PENNSYLVANIA.[1]

BY W. G. A. BONWILL, D.D.S., PHILADELPHIA.

(Continued from page 667.)

In Paris I was the guest of Dr. Amoedo, where for eight days I was in constant motion, not having a moment to spare either to enter any of the galleries of fine arts or to revel in the gayeties of this gayest of cities.

I saw much of Dr. Amoedo's practice, being frequently in his office. I was surprised to find him having so many antrum cases for treatment. I did not ask whether they were his originally. Whether he was successful in the treatment I could not ascertain. I should not want such a practice from others, nor would I wish to think they had resulted from my own bad dental operations. To me the number treated seemed large, as I have had but two cases in my practice in forty-three years, and those from the hands of others. His treatment was aseptic, and he had around him all the paraphernalia for the most complete carrying out of the mandates of Pasteur. I laughed at the doctor for wearing in his office and for every patient the same coat he wore upon the street. This is certainly objectionable, but he was not alone in this style of dressing.

While there an opportunity was given me to assist him in making a full set of plain teeth in my articulator, and which he, after several efforts, succeeded, to my delight, in accomplishing. I was pleased to help him, as that is the only way in which I can ever hope to teach any and all men this wonderful law of nature.

I have him to thank for marked attentions while at the Congress, and also all along the route from Moscow, he being familiar with three languages.

It was in Paris I came into contact with more American dentists than at any other place visited.

The profession here is split up into three sections and many cliques. The first to honor me was the French Odontological

---

[1] Read before the Odontological Society of Pennsylvania, March 12, 1898.

Society and School of Dentistry, with Professor Godon at its head. I lectured here to a crowded audience, on a very warm night and in close apartments, but they held together for two hours or more. Both Drs. Bogue and Amoedo acted as interpreters. The lecture included a synopsis of many of my methods of practice, and showing of special instruments. They especially desired an explanation of articulation by the large charts which I had brought with me from home.

Frenchman-like, many questions were asked, and it was evident that they knew something of my system and methods. The French dentist is full of energy and soul, and, while they are handicapped by their patients' desire for painless operations and plastic fillings, they are men of culture and standing, and hold their profession in high respect; and they move in social circles where ability is recognized, even though the dentist has to use his hands to obtain his living.

Taking Dr. Lecaudey as a true type of a man and dentist, we find him a member of the Legion of Honor, and through his intervention Dr. Michaels, an American dentist, was also made a member of the Legion. Dr. Lecaudey uses all his influence to forward socially and in every way the highest interests of the dental profession everywhere, and seems to be as much of a friend to the American dentists of Paris as to the French. He has always given me a dinner when in Paris, and shown his willingness to help in all possible directions.

The French School of Dentistry building was undergoing enlargement, and I was shown the drawings of the architect, whom I met when looking at the progress made. Unfortunately, they had made a mistake as to the light, which was easily demonstrated to them by placing a patient in a chair, and putting both in position to catch the light as they had arranged. The mistake was obviated at once.

The French medical dentists invited me to address them at Dr. Hugenschmidt's office the last Sunday I was in Paris, where Dr. Hugenschmidt acted as interpreter most satisfactorily; four hours were spent very pleasantly by me, and they exhibited their good breeding by close attention and every mark of respect.

Those holding the French D.D.S. and the French M.D., D.D.S. constitute two distinct societies and keep separate, although Dr. Hugenschmidt seems to be able to go to all the societies in Paris, being a man of extremely polite manners and marked intelligence, and careful of the feelings of others. I speak of him because I have known him so long, and his courtesy is always extended to me when in Paris. He is one of the moving spirits among the French M.D., D.D.S.

Dr. Godon is the most active member of the Odontological Society and School of Dentistry, and is an able and good fellow, and has his warm friends among the American element there. I cannot speak of each person individually, as time will not permit.

My last evening in Paris was spent with the American Dental Club, when they insisted upon my talking on every subject I am familiar with, consuming two hours or more. The discussion was voted over to the next monthly meeting, when the subject of laws of articulation was to be handled without gloves by Dr. Michaels and others. It required this and two subsequent meetings to finish the discussion.

A copy of the proceedings was sent to me, and Dr. Michaels seemed to take special delight in trying to tear the instrument to pieces, and said it was not amenable to any law, and he could do as well with a plaster articulator.

To sum up the opposition talk, I simply state what Dr. Bogue wrote me: "Bonwill, we took care of your laws and work, and, with all that could be said in criticism, you may rest assured you stand secure in your instrument's work and the laws underlying the mechanism of the human jaw."

The French and the Americans have always given me a good send-off, and I trust I may long keep their respect and love.

A pressing invitation was given me to attend the next (second) International Dental Congress in Paris in 1900, where at the same time the next International Medical Congress will meet; to which I replied that by that time, should I live and appear at the meeting, I hoped to tell them and show that I had entirely done away with gold as a filling-material in the human teeth, to which they all said, "Amen! so mote it be."

I found the amalgam question stirring the elements of each society, as well as the plastics, and, if possible, less gold-foil was being used if financial profit could be made by the change.

Cataphoresis was talked about, and should find a footing in Europe, and especially in Paris, since pain is so much dreaded, and it will no doubt be tried. But when the time consumed in the dental chair, which is as much dreaded as the operation, is taken into consideration, I think it will not stay very long. *I read them my paper on Cataphoresis, and how I had used the simple galvanic current from 1866 to 1869, and deadened sensitive dentine, extracted pulps,*

*and also teeth, but how a little incident, when using it one day, caused me to abandon it for something which I have now used with perfect satisfaction for thirty-five years.*

The laws regulating dentistry are growing more and more stringent, to the exclusion of the American dentist in France; and in every country in Europe the feeling grows apace to protect the native D.D.S. against any other country. It is only those who can stand a rigid examination and two years or more additional study in the schools of the kingdom or empire who can expect to remain or get into a European practice.

But it is right, as I said before, when we in every State have an Examining Board, and, no matter where graduated, must withstand their rigid examinations. In many respects this is fair, yet it works hardship against some who are competent to practise, but who are not able to answer the questions often put to them.

I know of a young student in this city who was thrown in his third year on such a foolish and irrelevant question as this: "What are the principal muscles engaged in the act of sneezing?" The young man was of the highest moral character and otherwise practically competent as an operator and in the laboratory.

We are forcing too many young men, such as they are, through our dental institutions, and the outlet, as heretofore, in foreign countries, will now be stopped, and we will have this half-educated element on our hands at home.

The eight days in Paris were completed, and, as I said, I had had no chance to have any fun. No one can leave this city without a regret that he has not seen everything in it.

As to the social side of my Paris visit, it was but a repetition of what had already been done for me, reviving the feeling that the labor had not been in vain, and that this special visit was not lost upon unappreciative audiences.

The meeting of the American Dental Club was at Dr. Bogue's, where, although late in the evening, a handsome dinner was given us. In this, as on all my former visits to Paris, Dr. Bogue has given me special consideration. Several of my old American friends were out of town. I saw much of Dr. Michaels. For some reason I could not make him feel I had anything new mechanically; he felt he had filled the whole bill. As to articulation, I was, in his opinion, far off the track. He did think I had a good thing in the dento-surgical engine, and went with me to be introduced to Professor Péan, the leading surgeon of France, and we took with us the surgical engine for his inspection.

They had in the hospital an apology for such an instrument, but, as I told them, it was only fit "for sawing wood." When Professor Péan had witnessed the work accomplished by my engine, he wanted one for his private and hospital work.

My stay was too short to enable me to visit many of the dentists at their private offices, yet I was shown every courtesy in a social way at lunches, dinners, etc., and in opportunity to examine their practice in the mouth.

I spent an evening with Dr. Michaels in reviewing some special work he had been doing in artificial limbs in connection with living tissue in supplying a substitute for the arm screwed into the living bone. He has been largely engaged in special hospital work, although he finds time to practise dentistry. He is very ingenious in this kind of work, but he was rather off his guard when he criticised my discovery of the laws of articulation. He was not up in mathematics or geometry as applied to the human frame, and, though famous as a member of the Legion of Honor, he showed in his criticism at the club meetings he was ill prepared to meet the question, and that there was more in the laws than he had calculated. "He is a good fellow for a' that," and I have him to thank for his close attention. The French are making every exertion to place their school on a higher plane, and to help to ennoble the profession and have its social status well recognized.

They are already planning for the Second International Dental Congress, which will meet here in 1900, at the same time the International Medical Congress will hold its sessions.

While the French dentist is not very fond of the American dentist, yet they extended to me every courtesy and considered me a cosmopolitan.

Any one can have a good opportunity for enjoyment in Paris if he is not too exclusive. We are as exclusive and selfish as any to be found anywhere in the ranks of professional men, and we have no cause to feel aggrieved at the treatment of the "American dentist" throughout Europe.

Dr. Thomas Evans was not looked upon as a dentist of a high type. It was his social intercourse with crowned heads that largely made his reputation. When I was in Paris in 1889 I called upon him. He refused to see me. His valet told me that as he did not use the machinery of modern dentistry, he would not see me because of my inventions. From what I could learn, he never operated other than by hand-pressure. Never having been in his office, I could not vouch for it, yet I believe it to be true.

It is largely true here, as all over Europe, that the French are very easily hurt. They will not tolerate pain nor an operation which inflicts it, and consequently the operator cannot have that chance to perform operations as his judgment would dictate, so he is at once handicapped. If cataphoresis can be of any avail, then it should go into use largely in Europe.

As to fees, there is no place abroad where higher prices are commanded, except, perhaps, in London. But their system of charges and making very short engagements with their patients must be abortive to that perfect system of practice which can only come through taking more time and longer sittings.

Until they are fully abreast with contour work we can never see dentistry advanced in the art of filling teeth. Gold crowns must come in to fill the breach, to the everlasting disgrace of *modern dentistry*, but the craze is no greater or wider spread than among our own "high-toned American dentistry."

Yet there *shall be a limit and a halt* to such false art and mechanism, for soon the day *will dawn* when it will be among "the things that were,—a school-boy's tale, the wonder of an hour;" when anticipation of decay, closer watchfulness to its limitation, the preparation of cavities to prevent recurrence, less gold, more plastics and the better how to use them than is done at present, and, not least, the *abnegation of self* and charge not for the material used, but for the *brains* put into it. In other words, *charge for a gutta-percha filling the same as for gold if it will be the safer.* Do this, and *all will be elevated!*

(To be continued.)

